# Vaccination with Astragalus and Ginseng Polysaccharides Improves Immune Response of Chickens against H5N1 Avian Influenza Virus

**DOI:** 10.1155/2016/1510264

**Published:** 2016-08-15

**Authors:** Auwalu Yusuf Abdullahi, Sanpha Kallon, Xingang Yu, Yongliang Zhang, Guoqing Li

**Affiliations:** ^1^Guangdong Provincial Zoonosis Prevention and Control Key Laboratory, College of Veterinary Medicine, South China Agricultural University, Guangzhou 510542, China; ^2^Animal Science Department, Kano University of Science and Technology Wudil, PMB 3244, Kano 20027, Nigeria; ^3^Animal Science Department, Njala University, Freetown, Sierra Leone; ^4^College of Animal Science, South China Agricultural University, Guangzhou 510542, China

## Abstract

To determine the effect of astragalus and ginseng polysaccharides (APS, GPS) on immune response and improvement of H5N1 vaccine, 360-day-old broilers were randomly divided into 8 groups of 45 chicks, comprising APS groups (1–3); GPS groups (4–6); vaccine group (7); and blank control (8) (without polysaccharide and vaccine). From day 12 after hatch groups 1–3 were given APS and groups 4–6 with GPS both at 100, 200, and 400 (mg/kg), respectively. At day 15 after hatch, groups 1–7 were vaccinated with 0.3 mL H5N1 vaccine subcutaneously; daily weight gain (DWG) and serum Ig antibody (by HI-test) were measured on 3, 7, 14, and 28 days after vaccination. Serum antibody titers and expression of cytokines (IL-2, IL-10, I FN-*γ*, and TNF) were determined by ELISA and RT-PCR. Results revealed that all the polysaccharide groups were numerically increased in antibody levels and the expression of cytokines was significant (*P* < 0.05) in the APS and GPS groups compared to corresponding vaccine group and blank control. DWG was higher (*P* < 0.05) in 400 mg/kg APS groups than control groups. Thus oral supplements of GPS and APS have shown their potential in the improvement of immune response and could be used as adjuvant in a formulation of H5N1 vaccine.

## 1. Introduction

H5N1 is an avian (bird) flu virus that has caused outbreaks in domestic poultry in parts of Asia and the Middle East. As H5N1 is so deadly to poultry, it is considered “highly pathogenic.” Since 2006, nearly 600 human infections with highly pathogenic H5N1 viruses have been reported to the World Health Organization (WHO) from 15 countries in Asia, Europe, and the Middle Near East. About 60% of these people died from this illness [1]. The first avian influenza virus to infect humans occurred in Hong Kong in 1997. The epidemic was linked to chickens and classified as avian influenza A (H5N1). Infected birds can shed influenza virus in their saliva, nasal secretions, and feces. Susceptible birds become infected when they have contact with contaminated secretions or excretions or with surfaces that are contaminated with secretions or excretion from infected birds [2].

The availability and use of effective vaccines can be a valuable tool in controlling outbreaks of avian influenza. Adjuvant killed vaccines can provide a strong humoral response and have been proved to be effective in preventing disease from mildly pathogenic avian influenza (MPAI) and highly pathogenic avian influenza (HPAI) challenges [3]. The HPAI virus is highly lethal in poultry and can cause large outbreaks leading to substantial economic loss and can spread directly from poultry to human, constituting a possible “pandemic threat” to the human population [4].

Astragalus polysaccharide (APS) possesses main components such as mannose, D-glucose, D-galactose, xylose, and L-arabinose. This polysaccharide is used as an immunomodulating agent in mixed herbal decoctions to treat common cold, diarrhea, fatigue, and anorexia [5]. It can also stimulate cell proliferation, induce the expression of surface antigens on lymphocytes, and affect the expression of cytokines and promote the production of antibodies [6]. Panax ginseng polysaccharide (GPS), on the other hand, contains several components such as ginsenosides, essential oil, peptidoglycans, polysaccharides, nitrogen-containing compounds, fatty acid, and phenolic compounds [7, 8]. It is well-known traditional Chinese herbal medicine. Logical studies have shown that GPS had multifunctions such as promoting the production of cytotoxic cells against tumors and stimulated macrophages to produce helper types 1 and 2 (Th1 and Th2) cytokines [9, 10]. GPS was also shown to modulate the antioxidant defense system such as superoxide dismutase and glutathione peroxidase probably via inducing regulatory cytokines [11, 12]. As anti-inflammatory responses at an early phase it results in the enhancement of antimicrobial activities and protection of mice from staphylococcus aureus-induced sepsis [13, 14]. Our previous work with APS and GPS on H9N2 indicated that APS treatment reduced H9N2 AIV replication; GPS was enhanced by pretreatment of CEF, and both have promoted early humoral immune responses in young chickens [15, 16].

In this research, we tested vaccinated and nonvaccinated chickens with H5N1 vaccine to evaluate the immunoregulatory effect of APS and GPS on chickens and assess the immunization potential of APS and GPS against H5N1 avian influenza.

## 2. Materials and Methods

### 2.1. Ethics

All animal experiments and husbandry involved in this study were treated in accordance with the guidelines of the South China Agricultural University Animal Care and Use Committee, which operates under the Animal Welfare Law and Regulations of the Department of Health and Human Services. The South China Agricultural University Animal Care and Use Committee has approved all protocols of this study.

### 2.2. APS and GPS

APS was bought from MEDICASS Company in Beijing China and GPS was provided by the Animal Science College of South China Agricultural University. GPS was extracted and purified as described in our previous works [15, 16]. Trizol, isopropanol, chloroform [trichloromethane], DEPC water, and 75% ethanol were used in the extraction of RNA. DNase1 [5 u/*μ*L], DNase1 buffer [10x], RNase inhibitor, agarose powder, and ethidium bromide were the other reagents used.

### 2.3. Purification of the Polysaccharides

The polysaccharides were purified as follows: removal of protein and pigment by salvage method [17] and active carbon adsorption and then through D101 macroaperture resin column, ADS-7 polymer adsorbents column, A-25 DEAE cellulose, and G-75 Sephadex column [18]. The polysaccharide contents (%) of APS and GPS were measured by vitriol-anthrone [17] taking anhydrous glucose as standard control. The percentage content (%) of APS and GPS were 79.50 and 75, respectively. For details refer to our previous works [15, 16].

### 2.4. Vaccine

The inactive avian influenza vaccine (H5N1 subtype) was obtained from South China Agricultural University Poultry Farm and 0.3 mL was injected subcutaneously on the dorsal region of the neck of 15-day-old chickens.

### 2.5. Experimental Design

Three hundred and sixty, 1-day-old Yue Huang avian broilers with an average weight of 60.5 g were randomly divided into 8 groups of 45 chicks each. They were housed in wire cages in air-conditioned room at 37°C and lighted for 24 h at the beginning of pretrial period. The temperature was gradually decreased to room temperature; lighting period was 12 hours per day. Chickens were fed with commercial starter diet, purchased from the university poultry farm. From day 12 after hatch groups 1–3 were supplemented orally with APS at 100 mg/kg, 200 mg/kg, and 400 mg/kg and groups 4–6 with GPS at 100 mg/kg, 200 mg/kg, and 400 mg/kg, respectively. Each dose of polysaccharide was dissolved in 1000 mL of water (till the end of the experiment). At 15 days after hatch, groups 1–7 were vaccinated with 0.3 mL H5N1 vaccine subcutaneously on the dorsal region of the neck, where group 7 only received vaccine without polysaccharide and group 8 was blank control without polysaccharide and vaccine. At 3, 7, 14, and 28 days after vaccination ten birds from each group (groups 1–8) were weighed before being euthanized by cervical dislocation; then blood samples were collected to separate serum and stored at −20°C until use. At the same time the spleen was weighed and immediately stored in liquid nitrogen. ELISA and RT-PCR tests were performed to determine the percentage of serum antibody titers and expression of cytokines (IL-2, IL-10, I FN-*γ*, and TNF).

### 2.6. Determination of Growth Performance

Weight of each bird was measured on days 3, 7, 14, and 28 after vaccination and effect on weight gain was recorded. Average live body weight, total weight gain, and daily weight gain of different groups were compared.

### 2.7. Measurement of Antibody Titer

Antibody titer was measured by hemagglutination inhibition (HI) test [19]. The HI test was a standard beta test, using 4 hemagglutinating units of antigen in 96-well plates, where the test serum had been diluted twofold. HI endpoint titers were determined as the reciprocal of the highest serum dilution that produced complete inhibition of hemagglutination.

### 2.8. RNA Extraction

Samples of spleen from the various groups of chickens were frozen in liquid nitrogen. About 60 g to 80 g of the tissue were cut into smaller pieces with sterilized scissors and washed with PBS. One mL of Trizol (RNA-solv Reagent) was added and the pieces were grinded by a polytron grinder to homogenize the tissue. The solution was placed in dry ice for 2-3 min to prevent the degradation of the RNA. The mixture was harvested at 12000 rpm for 10 min at 4°С. This was done to precipitate the undegraded substances such as DNA, proteins, and lipids. A short break was taken at this point; thereafter the content was poured into a new tube and 200 *μ*L of trichloromethane (chloroform) was added in order to separate RNA from DNA and protein. The content was shaken for 15 s and harvested at 12000 rpm for 15 min at 4°С. The aqueous upper phase (colorless liquid) was transferred in a new tube and 500 *μ*L of isopropanol was added and shaken for few seconds. The isopropanol was used to precipitate the RNA and the content was incubated at −20°С for 30 min and centrifuged at 12000 rpm for 10 min. RNA was precipitated and the supernatant was discarded leaving RNA pellet at the bottom of the tube. The tube was knocked slightly to displace the RNA and then washed with 1 mL of 75% ethanol. The content was harvested at 7500 rpm for 5 min at 4°С and the supernatant was discarded and the tube was placed upside down to dry the RNA for 5–10 min. After drying, the RNA pellet was dissolved in 50 *μ*L DEPC-treated H_2_O, vortexed and briefly centrifuged for 10 s.

### 2.9. Real-Time PCR

The total RNA was extracted from tissues using Trizol reagent (Takara Biotechnology, Dalian, China) for the detection of cytokines and H5N1 expression was tested by ultraviolet spectrophotometer at an optical density range of 1.8–2.0. The isolated RNA was digested with DNase1 (Takara Biotechnology, Dalian, China) at 37°C for 30 min. One (1) *μ*g of total RNA was used for reverse transcript with Rever Tra Ace QPCR RT kit (Toyobo Osaka, Japan) and amplifications were performed with 1 *μ*L cDNA in a total volume of 20 *μ*L. SYBR Green Real-Time PCR Master Mix (Roch Mortlake, Australia) was conducted with the Strata gene Mx3005P QPCR system (Strata gene) according to the manufacturer's instruction. All reactions were done in triplicate. Relative expression fold change was calculated by 2^−ΔΔCt^ method and *β*-actin was used as the endogenous reference gene to normalize the expression level of target gene. The primers used in the RT-PCR were listed in Table 1.

### 2.10. Detection of Interleukins (IL-2 and IL-10) in Chicken's Serum by ELISA

The ELISA Kits were purchased from Kemeidongya Biotechnology Company, Beijing, China, and were used to assay the levels of chicken interleukin 2 and interleukin 10 according to the manufacturer's instructions. The standard solutions were prepared from an original standard (640 ng/L) by serial dilution. Then the standard solutions (50 *μ*L/well) were pipetted into the wells of the first row of the microelisa striplate leaving the first two wells blank and serum sample (40 *μ*L/well) was pipetted into the remaining wells (two wells/group sample). Biotin-interleukin antibody (10 *μ*L) was added followed by Str-HRP-Conjugate Reagent (50 *μ*L/well). The microelisa striplate was sealed with closure plate membrane, shaken slightly, and incubated for 1 h at 37°С. Thereafter, the closure membrane was removed and liquid was drained and microelisa plate was washed five times by filling each well with wash buffer (350 *μ*L) using a squirt bottle, multichannel pipette, manifold dispenser, or auto washer. After the last wash, the remaining wash buffer was removed by aspirating or decanting and the plate was invert and blotted against clean paper towels. Chromogen solution A (50 *μ*L/well) was pipetted to each well followed by Chromogen B (50 *μ*L) which was protected from light to avoid light sensitive and thereafter it was incubated for 10 min at 37°С. Stop solution (50 *μ*L) was quickly added into each well to stop the reaction which was indicated by an instant change of blue to yellow color.

Taking the blank well as zero, measurement of the optical density (OD) under 450 nm wavelength was carried out within 10 min after adding the stop solution. According to the standard concentration and the corresponding OD values, the standard curve linear regression equation was calculated and then the OD values of the sample were applied on the regression equation to calculate the corresponding sample's concentration.

### 2.11. Statistical Analysis

All data were shown as mean ± SEM. Comparisons between two groups were analyzed using unpaired Student's* t*-tests and among multiple groups by ANOVA followed by a post hoc analysis using the Turkey's multiple comparison test using SPSS 17.0 software (SPSS, Chicago, IL, USA). A probability values (*P* < 0.05) was considered to be statistically significant. All experiments were performed at least three times.

## 3. Results

### 3.1. Differences in Body Weight

Differences in weight gain during experimental period were shown in Table 2. The weight differences of the various groups were recorded on days 3, 7, 14, and 28. There was an increase in weight gain on days 7, 14, and 28 by all the groups and the weight gains of the experimental groups were significant (*P* < 0.05). On days 7 and 14 the APS groups of 200 mg/kg and 400 mg/kg were higher than the corresponding groups, while on day 28 the body weight of APS (400 mg/kg) group was higher than corresponding GPS groups including vaccine and blank control. On day 3 there was no significant difference in body weight gain between the APS and GPS groups (*P* > 0.05). Hence, APS group of 400 mg/kg was higher (*P* < 0.05) than all at 7, 14, and 28 days.

### 3.2. Changes in Serum Antibody Titer

**Figure 1:**
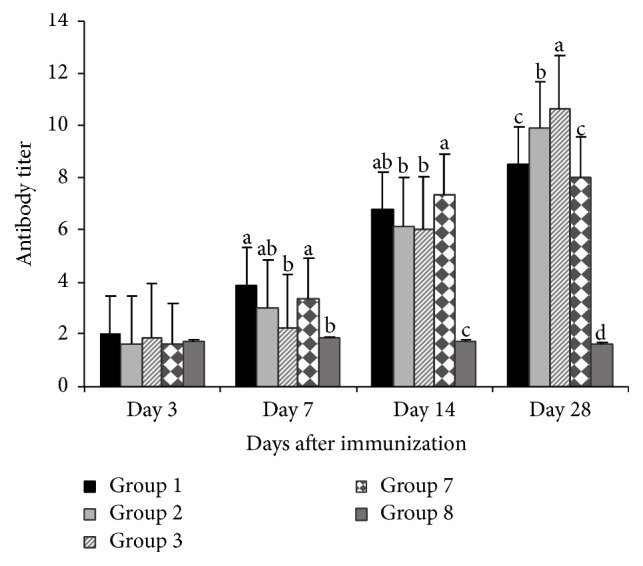
Antibody titers of APS treated groups and control groups. Superscripts with different letters (a–d) differ significantly (*P* < 0.05). Group 1 = 100 mg/kg APS + vaccine. Group 2 = 200 mg/kg APS + vaccine. Group 3 = 400 mg/kg APS + vaccine. Group 7 = vaccine only. Group 8 = no vaccine, no APS (blank control).

**Figure 2:**
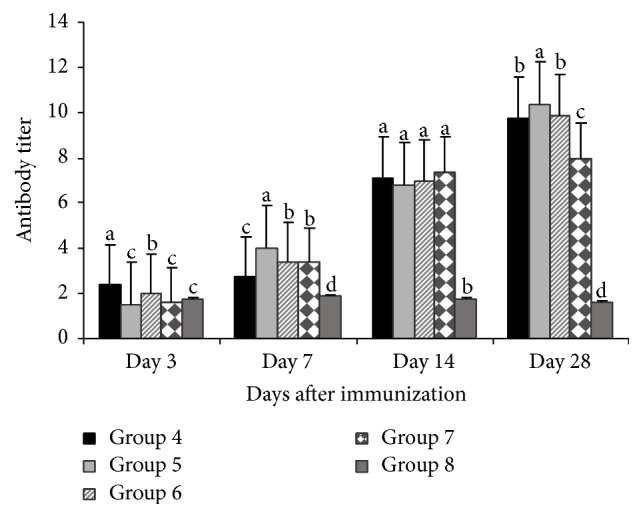
Antibody titers of GPS treated groups and control groups. Superscripts with different letters (a–d) differ significantly (*P* < 0.05). Group 4 = 100 mg/kg GPS + vaccine. Group 5 = 200 mg/kg GPS + vaccine. Group 6 = 400 mg/kg GPS + vaccine. Group 7 = vaccine only. Group 8 = no vaccine, no GPS (blank control).

Changes in serum antibody titers were shown in Figures 1 and 2. On day 3 after immunization, the antibody titer of all levels of APS treated groups was not different (*P* > 0.05) from vaccine group and blank control. But on days 7, 14, and 28 after vaccination APS were significantly different (*P* < 0.05) from corresponding nonpolysaccharide treated groups. On day 28 the antibody titer level of 400 mg/kg APS treated group was higher (*P* < 0.01) than vaccine group and blank control. However, it was observed that on days 14 and 28 the antibody titer level of blank control was significantly lower (*P* < 0.01) than vaccine group (Figure 1).

For GPS on day 3, after immunization the antibody titer level of 100 mg/kg GPS treated group was higher (*P* < 0.05) than vaccine group and blank control. Similarly on day 7, the antibody titer levels of the 200 mg/kg GPS treated group was higher (*P* < 0.05) than vaccine group and blank control. On day 28 the antibody titer level of 200 mg/kg GPS treated group was significantly higher (*P* < 0.01) than vaccine and blank control, while the antibody titer levels of the 100 mg/kg and 400 mg/kg GPS treated groups were higher (*P* < 0.05) than vaccine group and blank control. However, on days 14 and 28 the antibody titer level of the blank control was significantly very low (*P* > 0.01), while on day 7 it was significantly lower (*P* > 0.05) than vaccine group (Figure 2).

### 3.3. Expression of Cytokines

**Figure 3:**
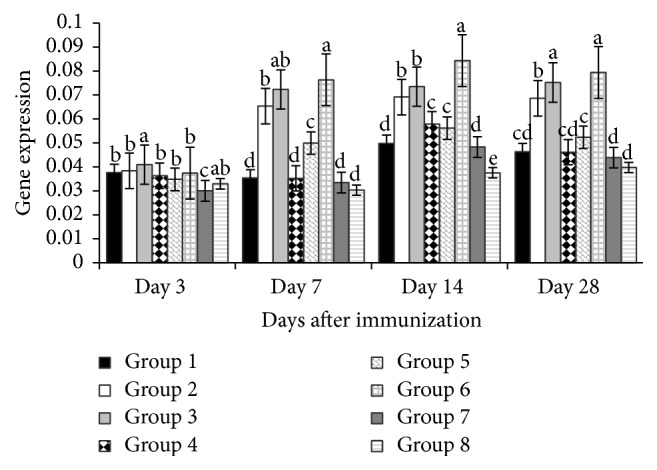
Effect of APS and GPS on gene expression of TNF-*α*. Superscripts with different letters (a–e) differ significantly (*P* < 0.05). Group 1 = 100 mg/kg APS + vaccine. Group 2 = 200 mg/kg APS + vaccine. Group 3 = 400 mg/kg APS + vaccine. Group 4 = 100 mg/kg GPS + vaccine. Group 5 = 200 mg/kg GPS + vaccine. Group 6 = 400 mg/kg GPS + vaccine. Group 7 = vaccine only. Group 8 = no vaccine, no GPS (blank control).

Figure 3 revealed that on day 3 after vaccination the TNF-*α* gene expressions of the polysaccharide treated groups (all levels of APS and 100 mg/kg and 400 mg/kg GPS) were higher (*P* < 0.05) than corresponding nonpolysaccharide treated groups, while, on day 7, the gene expressions of polysaccharide treated groups (200 mg/kg and 400 mg/kg of both APS and GPS) were higher (*P* < 0.01) than nonpolysaccharide treated groups. Similarly on days 14 and 28, the gene expressions of the polysaccharide treated groups (200 mg/kg and 400 mg/kg APS and 400 mg/kg GPS) were higher (*P* < 0.01) than corresponding nonpolysaccharide treated groups.

**Figure 4:**
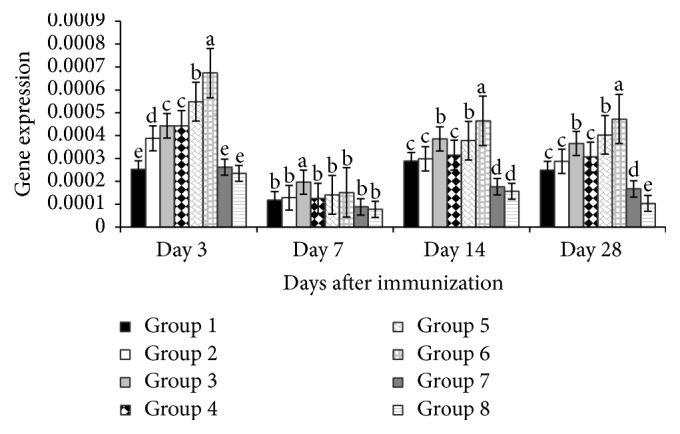
Effect of APS and GPS on gene expression of IFN-*β*. Superscripts with different letters (a–e) differ significantly (*P* < 0.05). Group 1 = 100 mg/kg APS + vaccine. Group 2 = 200 mg/kg APS + vaccine. Group 3 = 400 mg/kg APS + vaccine. Group 4 = 100 mg/kg GPS + vaccine. Group 5 = 200 mg/kg GPS + vaccine. Group 6 = 400 mg/kg GPS + vaccine. Group 7 = vaccine only. Group 8 = no vaccine, no GPS (blank control).

Figure 4 depicted that on day 3 the IFN-*β* gene expressions of the experimental groups (200 mg/kg and 400 mg/kg APS; and all levels of GPS) were higher (*P* < 0.01) than vaccine group and blank control. Equally, on day 7 the gene expression of the 400 mg/kg APS treated group was higher (*P* < 0.01), while both APS and GPS group at 200 mg/kg were significant (*P* < 0.05) compared with vaccine group and blank control. Furthermore on days 14 and 28 after vaccination, the gene expressions of the polysaccharide treated groups, all levels of both APS and GPS were higher (*P* < 0.01) than nonpolysaccharide treated groups.

**Figure 5:**
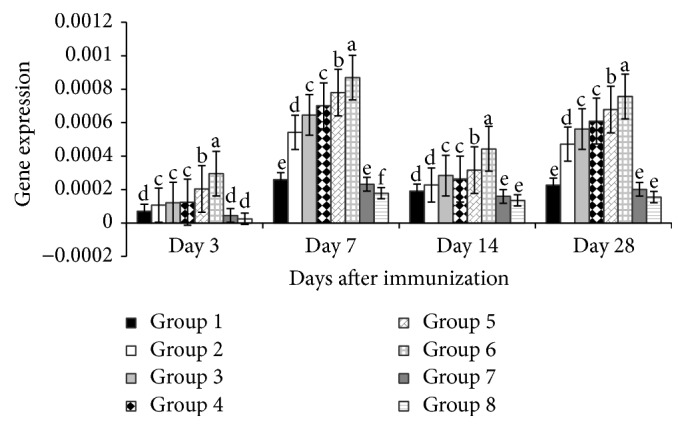
Effect of APS and GPS on IL-2 gene expression. Superscripts with different letters (a–f) differ significantly (*P* < 0.05). Group 1 = 100 mg/kg APS + vaccine. Group 2 = 200 mg/kg APS + vaccine. Group 3 = 400 mg/kg APS + vaccine. Group 4 = 100 mg/kg GPS + vaccine. Group 5 = 200 mg/kg GPS + vaccine. Group 6 = 400 mg/kg GPS + vaccine. Group 7 = vaccine only. Group 8 = no vaccine, no GPS (blank control).

Figure 5 illustrated that, on day 3, the IL-2 gene expression of the 200 mg/kg and 400 mg/kg GPS treated groups were higher (*P* < 0.01), while the 200 mg/kg and 400 mg/kg APS and the 100 mg/kg GPS treated groups were higher (*P* < 0.05) than vaccine group and blank control. We observed that on days 3, 7, and 28 the gene expressions of all levels of GPS treated groups were higher (*P* < 0.05) than the corresponding APS groups. And on days 7, 14, and 28 IL-2 gene expressions of the 400 mg/kg APS and GPS treated groups were higher (*P* < 0.01); likewise on days 7 and 28, the IL-2 gene expressions of APS and GPS treated groups have shown similar trend.

**Figure 6:**
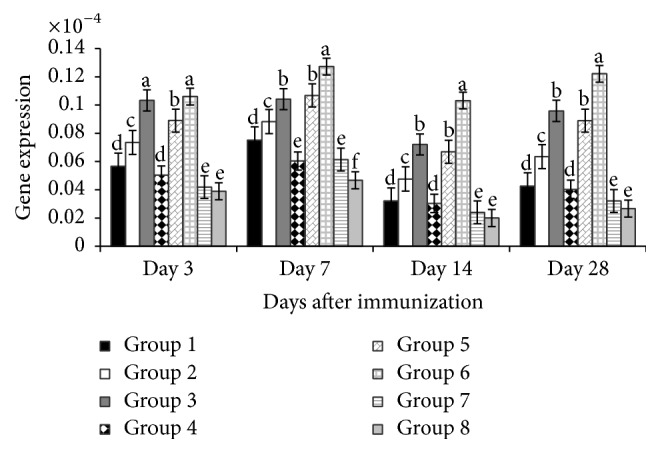
Effect of APS and GPS on gene expression of IL-10. Superscripts with different letters (a–f) differ significantly (*P* < 0.05). Group 1 = 100 mg/kg APS + vaccine. Group 2 = 200 mg/kg APS + vaccine. Group 3 = 400 mg/kg APS + vaccine. Group 4 = 100 mg/kg GPS + vaccine. Group 5 = 200 mg/kg GPS + vaccine. Group 6 = 400 mg/kg GPS + vaccine. Group 7 = vaccine only. Group 8 = no vaccine, no GPS (blank control).

Figure 6 showed that, on days 3, 7, 14, and 28 after immunization, the IL-10 gene expressions of both 200 mg/kg and 400 mg/kg of APS and GPS treated groups were higher (*P* < 0.01) than vaccine group and blank control, while the IL-10 gene expressions of the 100 mg/kg APS and GPS were higher (*P* < 0.05) than vaccine group and blank control. On days 3, 7, 14, and 28 the IL-10 gene expressions of the 200 mg/kg and 400 mg/kg GPS treated groups were higher (*P* < 0.05) than corresponding 200 mg/kg and 400 mg/kg APS treated groups.

### 3.4. Interleukins (IL-2 and IL-10) in Chicken's Serum by ELISA

**Figure 7:**
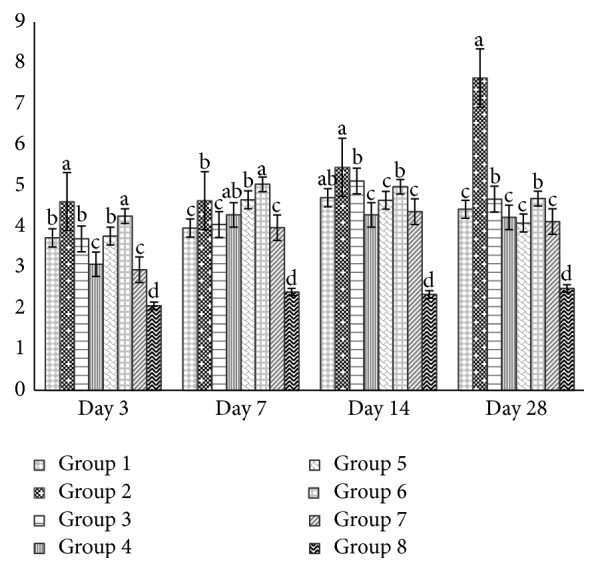
Effect of APS and GPS on IL-2 by ELISA. Superscripts with different letters (a–d) differ significantly (*P* < 0.05). Group 1 = 100 mg/kg APS + vaccine. Group 2 = 200 mg/kg APS + vaccine. Group 3 = 400 mg/kg APS + vaccine. Group 4 = 100 mg/kg GPS + vaccine. Group 5 = 200 mg/kg GPS + vaccine. Group 6 = 400 mg/kg GPS + vaccine. Group 7 = vaccine only. Group 8 = no vaccine, no GPS (blank control).

**Figure 8:**
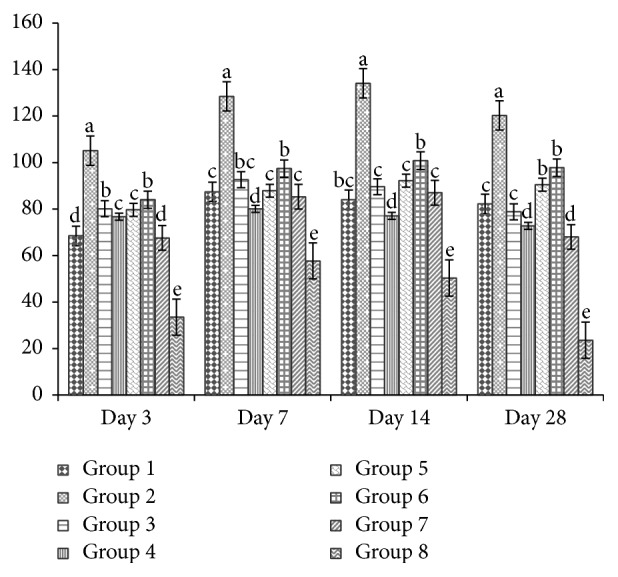
Effect of APS and GPS on IL-10 by ELISA. Superscripts with different letters (a–e) differ significantly (*P* < 0.05). Group 1 = 100 mg/kg APS + vaccine. Group 2 = 200 mg/kg APS + vaccine. Group 3 = 400 mg/kg APS + vaccine. Group 4 = 100 mg/kg GPS + vaccine. Group 5 = 200 mg/kg GPS + vaccine. Group 6 = 400 mg/kg GPS + vaccine. Group 7 = vaccine only. Group 8 = no vaccine, no GPS (blank control).

Figures 7 and 8 revealed ELISA results for IL-2 and IL-10 and very high levels were produced after H5N1 vaccination. On days 3, 7, 14, and 28 both IL-2 and IL-10 of APS at 200 mg/kg and 400 mg/kg of GPS groups were higher (*P* < 0.01) than vaccine group and blank control.

## 4. Discussion 

The present study demonstrated that the combination of APS or GPS with H5N1 vaccine may have better protective effects against lethal H5N1 influenza virus infection in chickens. It was previously reported that APS and other Chinese herbs had adjuvant effect when coadministered with an influenza vaccine, and it increased both the innate and systemic humoral responses that provide complex protection against H5N1 in chickens [20]. In this study dynamic changes in body weight were observed on day 28 in which the experimental groups (400 mg/kg APS and GPS) were higher than vaccine group and blank control, probably due to better health situation of chickens (Table 2). The results further revealed that administration of GPS and APS was able to protect the chickens from viral damage after vaccination. This is in parallel with Hu [21] who reported that animals treated with Chinese herbal medicine or Chinese herbal ingredients before or after vaccination showed a reduced incidence of infectious diseases and an increased immune response. However, no significant difference in body weight gain was observed on day 3 in both GPS and APS treated groups (Table 2).

Serum HI Ab titer is a valid indicator of the humoral immunity in chickens [22]. Previous studies have shown that the HI Ab is directly effective against NDV in chickens [23, 24]. Our result showed that the HI Ab titers in the treatment groups of GPS and APS were significantly higher than vaccine group after immunization (Figures 1 and 2), suggesting that GPS and APS could enhance the humoral immunity. It demonstrated that, on days 14 and 28 after vaccination, the anti-H5N1 HI Ab titers of the APS treated groups (200 mg/kg and 400 mg/kg) and 100 mg/kg, 200 mg/kg, and 400 mg/kg GPS treated groups were higher than vaccine group and blank control, indicating a more rapid response and potent effects in increasing the production of anti-H5N1 antibodies of the GPS and APS treatment groups. These findings provide evidence for the use of GPS and APS as effective herbal medicinal immune stimulators.

More interestingly, vaccination of H5N1 caused decrease in body weight as shown in the results, but addition of both GPS and APS was able to recover the decreased body weights, probably due to body regulation to vaccination stress. The effects of APS and GPS on body weight gain were different, especially the doses from 200 mg/kg to 400 mg/kg; it is fascinating that body weight gain was always lower using GPS rather than APS, probably due to their chemical components, although both contain polysaccharides as their constituents but the latter contains more simple sugars than the former, which might contribute to the effects. Refer to Ríos and Waterman [25] for detailed review on astragalus.

The reactivity of chicken spleen to antigens is higher in magnitude with increase in age [26]. The spleen constitutively expressed IL-4, IL-10, and IFN-*γ* cytokine genes as early as embryonic day 12 which is associated with shaping the spleen environment. The expression patterns of these cytokines coincide with the completion of colonization of the spleen by cellular migrants from the thymus [26]. In this study we recorded that on days 7, 14, and 28 the TNF-*α* gene expression in the spleen of the APS and GSP (200 mg/kg and 400 mg/kg) treated groups was higher than corresponding vaccine group and blank control. TNF involves in a network of cytokines and chemokines that stimulates the recruitment of immune cells in the infectious foci and can block the viral replication by interfering with the viral life cycle especially in viral entry [27].

Previous reports found APS and GPS to promote IL-2 bioactivity [28]. Accordingly, the increased bioactivity of IL-2 of peripheral blood lymphocytes may partly explain the increased lymphocytes proliferation. The Th2 cells secrete cytokines such as IL-4 and IL-10 which help B cell proliferation and are associated with humoral immunity [28]. In our finding it was revealed that gene expressions of IL-2 and IL-10 in APS and GPS groups were evidently higher than vaccine group and control, meaning that APS and GPS most probably stimulate cytokine production. Cytokines often have multiple effects, which originated from various effector cells. The variation of cytokines in our study coincided with finding of Shao et al. [29]. In comparison there was no significant difference in body weight; however, the cytokine gene expressions in the GPS groups were significant compared with corresponding APS groups. Hence APS which had been known over the centuries for its immune potency can be substituted by a more immune potent GPS as adjuvant in the formulation of a H5N1 vaccine. Our results indicated that oral supplement of both GPS and APS will be beneficial to health and vaccination efficiency in H5N1 vaccine in chickens. More interestingly, GPS exerted similar and even better improvement in this respect.

## 5. Conclusion

Conclusively our results showed the following. (1) The addition of APS and GPS is beneficial to eliminate the chicken weight loss after vaccination. (2) APS and GPS have adjuvant properties to enhance the antibody level when used in combination with influenza vaccine (H5N1). (3) Expressions of IL-2, IL-10, and TNF-*α* and IFN-*β* in GPS and APS treated groups were higher than control group, suggesting GPS and APS stimulated cytokine production and macrophage activation. Intriguingly, expressions of IL-2 and IL-10 by ELISA have proved similar results. (4) The effects of GPS and APS on growth rate and immune response were related to their dosages used. Appropriate effective dosages such as 200 mg/kg and 400 mg/kg should be taken into consideration in the use of GPS and APS as adjuvant in the formulation of a new H5N1 vaccine. (5) The study further revealed that the combination of APS or GPS with H5N1 vaccine may have better protective effects against lethal H5N1 influenza virus infection in chickens.

Generally, oral supplementation of GPS and APS dosages (100 mg/kg, 200 mg/kg, and 400 mg/kg) was beneficial to the health of the chickens to eliminate the chicken weight loss after vaccination. Similarly doses of 200 mg/kg and 400 mg/kg GPS and APS in vivo would be expected to serve as adjuvant for new vaccine formulation against H5N1 AIV.

## Figures and Tables

**Table 1:** Sequence of the oligonucleotide primers used in Real-Time PCR.

Gene name	Primer (5′ → 3′)	Product (bp)	Accession number
IL-2	F: CTTTGGCTGTATTTCGG	163	NM-204153.1
	R: CTGGGTCTCAGTTGGTGT		

IL-10	F: GCTGAGGGTGAAGTTTGAG	192	NM-001004414.2
	R: TGATGACTGGTGCTGGTCT		

TNF-*α*	F: CTCAGGACAGCCTATGCCA	171	AY765397.1
	R: CACGACAGCCAAGTCAACG		

IFN-*β*	F: CATACTGAGCCAGATTGTTTCG	176	NM-205149.1
	R: TCAAGTCGTTCATCGGGAG		

*β*-actin	F: TGATATTGCTGCGCTCGTTG	202	JF436880.1
	R: CTTTCTGGCCCATACCAACC		

**Table 2:** Effect of APS and GPS on body weight gain (g).

Groups	D3	D7	D14	D28
APS (100 mg/kg)	117.8 ± 8.49	148.3 ± 11.59^b^	214.9 ± 12.44^c^	396.1 ± 21.31^b^
GPS (100 mg/kg)	107.2 ± 9.51	146.9 ± 13.03^b^	207.1 ± 20.6^c^	398.4 ± 23.82^b^
APS (200 mg/kg)	113.3 ± 10.99	150.6 ± 12.5^b^	240.5 ± 16.43^b^	397.8 ± 22.53^b^
GPS (200 mg/kg)	112.6 ± 8.9	145.7 ± 12.96^b^	213.4 ± 25.42^c^	371.5 ± 28.5^b^
APS (400 mg/kg)	119.2 ± 10.78	166.4 ± 13.01^a^	261.4 ± 18.7^a^	432.0 ± 23.65^a^
GPS (400 mg/kg)	117.6 ± 8.48	147.3 ± 14.15^b^	235.8 ± 22.73^b^	410.51 ± 27.34^b^
Vaccine	109.7 ± 9.72	139.7 ± 14.73^c^	197.5 ± 21.91^c^	382.2 ± 23.9^b^
Blank control	117.2 ± 9.07	146.8 ± 13.03^b^	235.8 ± 22.73^b^	382.8 ± 24.79^b^

Superscripts with different letters (a, b, and c) differ significantly (*P* < 0.05).

APS = astragalus polysaccharide; GPS = ginseng polysaccharide; D = day.
